# Global optimization in Hilbert space

**DOI:** 10.1007/s10107-017-1215-7

**Published:** 2017-12-16

**Authors:** Boris Houska, Benoît Chachuat

**Affiliations:** 1 School of Information Science and Technology, ShanghaiTech University, 319 Yueyang Road, Shanghai, 200031 China; 2 Department of Chemical Engineering, Centre for Process Systems Engineering, Imperial College London, South Kensington Campus, London, SW7 2AZ UK

**Keywords:** Infinite-dimensional optimization, Complete search, Branch-and-lift, Convergence analysis, Complexity analysis, 49M30, 65K10, 90C26, 93B40

## Abstract

We propose a complete-search algorithm for solving a class of non-convex, possibly infinite-dimensional, optimization problems to global optimality. We assume that the optimization variables are in a bounded subset of a Hilbert space, and we determine worst-case run-time bounds for the algorithm under certain regularity conditions of the cost functional and the constraint set. Because these run-time bounds are independent of the number of optimization variables and, in particular, are valid for optimization problems with infinitely many optimization variables, we prove that the algorithm converges to an $\varepsilon$-suboptimal global solution within finite run-time for any given termination tolerance $\varepsilon > 0$. Finally, we illustrate these results for a problem of calculus of variations.

## Introduction

Infinite-dimensional optimization problems arise in many research fields, including optimal control [7, 8, 24, 54], optimization with partial differential equations (PDE) embedded [22], and shape/topology optimization [5]. In practice, these problems are often solved approximately by applying discretization techniques; the original infinite-dimensional problem is replaced by a finite-dimensional approximation that can then be tackled using standard optimization techniques. However, the resulting discretized optimization problems may comprise a large number of optimization variables, which grows unbounded as the accuracy of the approximation is refined. Unfortunately, worst-case run-time bounds for complete-search algorithms in nonlinear programming (NLP) scale poorly with the number of optimization variables. For instance, the worst-case run-time of spatial branch-and-bound [17, 44] scales exponentially with the number of optimization variables. In contrast, algorithms for solving convex optimization problems in polynomial run-time are known [11, 40], e.g. in linear programming (LP) or convex quadratic programming (QP). While these efficient algorithms enable the solution of very large-scale convex optimization problems, such as structured or sparse problems, in general their worst-case run-time bounds also grow unbounded as the number of decision variables tends to infinity.

Existing theory and algorithms that directly analyze and exploit the infinite-dimensional nature of an optimization problem are mainly found in the field of convex optimization. For the most part, these algorithms rely on duality in convex optimization in order to construct upper and lower bounds on the optimal solution value, although establishing strong duality in infinite-dimensional problems can prove difficult. In this context, infinite-dimensional linear programming problems have been analyzed thoroughly [3]. A variety of algorithms are also available for dealing with convex infinite-dimensional optimization problems, some of which have been analyzed in generic Banach spaces [14], as well as certain tailored algorithms for continuous linear programming [4, 13, 32].

In the field of non-convex optimization, problems with an infinite number of variables are typically studied in a local neighborhood of a stationary point. For instance, local optimality in continuous-time optimal control problems can be analyzed by using Pontryagin’s maximum principle [46], and a number of local optimal control algorithms are based on this analysis [6, 12, 51, 54]. More generally, approaches in the classical field of variational analysis [37] rely on local analysis concepts, from which information about global extrema may not be derived in general. In fact, non-convex infinite-dimensional optimization remains an open field of research and, to the best of our knowledge, there currently are no generic complete-search algorithms for solving such problems to global optimality.

This paper asks the question whether a global optimization algorithm can be constructed, whose worst-case run-time complexity is independent of the number of optimization variables thereof, such that this algorithm would remain tractable for infinite-dimensional optimization problems. Clearly, devising such an algorithm may only be possible for a certain class of optimization problems. Interestingly, the fact that the “complexity” or “hardness” of an optimization problem does not necessarily depend on the number of optimization variables has been observed—and it is in fact exploited—in state-of-the-art global optimization solvers for NLP/MINLP, although these observations are still to be analyzed in full detail. For instance, instead of applying a branch-and-bound algorithm in the original space of optimization variables, global NLP/MINLP solvers such as BARON [49, 52] or ANTIGONE [34] proceed by lifting the problem to a higher-dimensional space via the introduction of auxiliary variables from the DAG decomposition of the objective and constraint functions. In a different context, the solution of a lifted problem in a higher-dimensional space has become popular in numerical optimal control, where the so-called multiple-shooting methods often outperform their single-shooting counterparts despite the fact that the former calls for the solution a larger-scale (discretized) NLP problem [7, 8]. This idea that certain optimization problems become easier to solve than equivalent problems in fewer variables is also central to the work on lifted Newton methods [2]. To the best of our knowledge, such behaviors cannot be explained currently with results from the field of complexity analysis, which typically give monotonically increasing worst-case run-time bounds as the number of optimization variables increases. In this respect, these run-time bounds therefore predict the opposite behavior to what can sometimes be observed in practice.

### Problem formulation

The focus of this paper is on complete-search algorithms for solving non-convex optimization problems of the form:

1 $$\begin{aligned} \inf _{x \in C} \; F(x) \,, \end{aligned}$$

where $F: H \rightarrow \mathbb R$ and $C \subseteq H$ denote the cost functional and the constraint set, respectively; and the domain *H* of this problem is a (possibly infinite-dimensional) Hilbert space with respect to the inner product $\langle \cdot ,\cdot \rangle : H \times H \rightarrow \mathbb R$. The theoretical considerations in the paper do not assume a separable Hilbert space, although our various illustrating examples are based on separable spaces.

#### Definition 1

A feasible point $x^* \in C$ is said to be an $\varepsilon$-suboptimal global solution—or $\varepsilon$-global optimum–of (1), with $\varepsilon > 0$, if

$$\begin{aligned} \forall x \in C, \quad F(x^*)\ {\le }\ F(x) + \varepsilon \,. \end{aligned}$$

We make the following assumptions regarding the geometry of *C* throughout the paper.

#### Assumption 1

The constraint set *C* is convex, has a nonempty relative interior, and is bounded with respect to the induced norm on *H*; that is, there exists a constant $\gamma <\infty$ such that

$$\begin{aligned} \forall x \in C, \quad \Vert x \Vert _H := \sqrt{\langle x,x \rangle } \le \gamma \,. \end{aligned}$$

Our main objective in this paper is to develop an algorithm that can locate an $\varepsilon$-suboptimal global optimum of Problem (1), in finite run-time for any given accuracy $\varepsilon >0$, provided that *F* satisfies certain regularity conditions alongside Assumption 1.

#### Remark 1

Certain infinite-dimensional optimization problems are formulated in a Banach space $(B,\Vert \cdot \Vert )$ rather than a Hilbert space, for instance in the field of optimal control of partial differential equations in order to analyze the existence of extrema [22]. The optimization problem (1) becomes

2 $$\begin{aligned} \inf _{x \in \hat{C}} \; \hat{F}(x) \end{aligned}$$

with $\hat{F}: B \rightarrow \mathbb R$ and $\hat{C}$ a convex bounded subset of *B*. Provided that:

1. the Hilbert space $H\subseteq B$ is convex and dense in $(B,\Vert \cdot \Vert )$;
2. the function $\hat{F}$ is upper semi-continuous in $\hat{C}$; and
3. the constraint set $\hat{C}$ has a nonempty relative interior;

we may nonetheless consider Problem (1) with $C := \hat{C} \cap H$ instead of (2), for any $\varepsilon$-suboptimal global solution of the former is also an $\varepsilon$-suboptimal global solution of (2), and both problems have such $\varepsilon$-suboptimal points. Because Conditions 1–3 are often satisfied in practical applications, it is for the purpose of this paper not restrictive to assume that the domain of the optimization variables is indeed a Hilbert space.

### Outline and contributions

The paper starts by discussing several regularity conditions for sets and functionals defined in a Hilbert space in Sect. 2, based on which complete-search algorithms can be constructed whose run-time is independent of the number of optimization variables. Such an algorithm is presented in Sect. 3 and analyzed in Sect. 4, which constitutes the main contributions and novelty. A numerical case study is presented in Sect. 5 in order to illustrate the main results, before concluding the paper in Sect. 6.

Although certain of these algorithmic ideas are inspired by a recent paper on global optimal control [25], we develop herein a much more general framework for optimization in Hilbert space. Besides, Sect. 4 derives novel worst-case complexity estimates for the proposed algorithm. We argue that these ideas could help lay the foundations towards new ways of analyzing the complexity of certain optimization problems based on their structural properties rather than their number of optimization variables. Although the run-time estimates for the proposed algorithm remain conservative, they indicate that complexity in numerical optimization does not necessarily depend on whether the problem at hand being small-scale, large-scale, or even infinite-dimensional.

## Some regularity conditions for sets and functionals in Hilbert space

This section builds upon basic concepts in infinite-dimensional Hilbert spaces in order to arrive at certain regularity conditions for sets and functionals defined in such spaces. Our focusing on Hilbert space is motivated by the ability to construct an orthogonal basis $\varPhi _0, \varPhi _1, \ldots \in H$ such that

$$\begin{aligned} \forall i,j \in \mathbb N, \quad \frac{1}{\sigma _i} \langle \varPhi _i , \varPhi _j \rangle \; = \; \delta _{i,j} \; := \; \left\{ \begin{array}{ll} 0 &{} \quad \text {if }i \ne j, \\ 1 &{} \quad \text {otherwise,} \end{array} \right. \end{aligned}$$

for some scalars $\sigma _0, \sigma _1, \ldots \in \mathbb R^{++}$. We make the following assumption throughout the paper:

### Assumption 2

The basis functions $\varPhi _k$ are uniformly bounded with respect to $\Vert \cdot \Vert _H$.

Equipped with such a basis, we can define the associated projection functions $P_M: H \rightarrow H$ for each $M \in \mathbb N$ as

$$\begin{aligned} \forall x \in H, \quad P_M(x) \; := \; \sum _{k=0}^M \frac{\langle x, \varPhi _k \rangle }{\sigma _k} \; \varPhi _k \; . \end{aligned}$$

A natural question to ask at this point, is what can be said about the distance between an element $x \in H$ and its projection $P_M(x)$ for a given $M\in \mathbb N$.

### Definition 2

We call $D(M,x) \; := \; \left\| \, x - P_M(x) \, \right\| _H$ the distance between an element $x \in H$ and its projection $P_M(x)$. Moreover, given the constraint set $C\subseteq H$, we define

$$\begin{aligned} \overline{D}_C(M) \; := \; \sup _{x \in C} \, D(M,x) \;. \end{aligned}$$

### Lemma 1

Under Assumption 1, the function $\overline{D}_C:\mathbb N \rightarrow \mathbb R$ is uniformly bounded from above by $\gamma$.

### Proof

For each $M\in \mathbb N$, we have

$$\begin{aligned} \left[ \overline{D}_C(M) \right] ^2= & {} \sup _{x \in C} \, \left\| \, x - P_M(x) \, \right\| _H^2 \; \le \; \left\| \, x \, \right\| _H^2 \; . \end{aligned}$$

The result follows by Assumption 1. $\square$

Despite being uniformly bounded, the function $\overline{D}_C(M)$ may not converge to zero as $M \rightarrow \infty$ in an infinite-dimensional Hilbert space in general. Such lack of convergence is illustrated in the following example.

### Example 1

Consider the case that all the basis functions $\varPhi _0,\varPhi _1,\ldots$ are in the constraint set *C*, and define the sequence $\{x_k\}_{k\in \mathbb N}$ with $x_k:=\varPhi _{k+1}$. For all $k\in \mathbb N$, we have $P_k(x_k)=0$, and therefore

$$\begin{aligned} \overline{D}_C(k) \;\ge \; D(k,x_k) \;=\; \left\| \, x_k - P_k(x_k) \, \right\| _H = \left\| \, x_k \, \right\| _H \; = \; 1 \,. \end{aligned}$$

$$\square $$

This behavior is unfortunate because the existence of minimizers to Problem (1) cannot be ascertained without making further regularity assumptions. Moreover, for a sequence $(x_k)_{k \in \mathbb N}$ of feasible points of Problem (1) converging to an infimum, it could be that

$$\begin{aligned} \limsup _{M \rightarrow \infty } \, \limsup _{k \rightarrow \infty } \, D(M,x_k) \; \ne \; \limsup _{k \rightarrow \infty } \, \limsup _{M \rightarrow \infty } \, D(M,x_k) \; . \end{aligned}$$

That is, any attempt to approximate the infimum by constructing a sequence of finite parameterizations of the optimization variable *x* could in principle be unsuccessful.

A principal aim of the following sections is to develop an optimization algorithm, whose convergence to an $\varepsilon$-global optimum of Problem (1) can be certified. But instead of making assumptions about the existence, or even the regularity, of the minimizers of Problem (1), we shall impose suitable regularity conditions on the objective function *F* in (1). In preparation for this analysis, we start by formalizing a particular notion of regularity for the elements of *H*.

### Definition 3

An element $g \in H$ is said to be *regular for the constraint set C* if

3 $$\begin{aligned} \lim _{M \rightarrow \infty } R_C(M,g) \; = \; 0 \quad \text {with} \quad R_C(M,g) := \overline{D}_C(M) D(M,g) \; . \end{aligned}$$

Moreover, we call the function $R_C(\cdot ,g): \mathbb N \rightarrow \mathbb R^+$ the *convergence rate at g on C*.

### Theorem 1

For any $g \in H$, we have

4 $$\begin{aligned} \forall M\in \mathbb N, \quad&\sup _{x \in C} \; \left| \, \langle g , x-P_M(x) \rangle \, \right| \; \le \; R_C(M,g) \; . \end{aligned}$$

In the particular case of *g* being a regular element for *C*, we have

$$\begin{aligned} \lim _{M \rightarrow \infty } \; \sup _{x \in C} \; \left| \, \langle g , x-P_M(x) \rangle \, \right| \; = \; 0 \; . \end{aligned}$$

### Proof

Let $M\in \mathbb N$, and consider the optimization problem

$$\begin{aligned} \overline{V}_M \, := \, \sup _{x \in C} \; \langle g, x - P_M(x) \rangle \, = \, \sup _{x \in C} \; \langle g, w \rangle \; , \end{aligned}$$

where we have introduced the variable $w := x - P_M(x)$ such that

$$\begin{aligned} \forall x\in C,\quad \Vert w \Vert _H \, \le \, \overline{D}_C(M) \;. \end{aligned}$$

Since the functions $\varPhi _0, \ldots , \varPhi _M$ are orthogonal to each other, we have $\langle \varPhi _k , w \rangle = 0$ for all $k \in \{ 0, \ldots , M\}$, and it follows that

$$\begin{aligned} \overline{V}_M \, \le \, \sup _{w \in H} \; \langle g, w \rangle \quad \text {s.t.} \quad \langle \varPhi _0 , w \rangle \, = \, \cdots \, = \, \langle \varPhi _M , w \rangle \, = \, 0 \; , \quad \Vert w \Vert _H \, \le \, \overline{D}_C(M) \,. \end{aligned}$$

Next, we use duality to obtain

$$\begin{aligned} \overline{V}_M \, \le \, \inf _{\lambda \in \mathbb R^{M+1}} \; \sup _{w \in H} \; \left\langle \, g - \sum _{k=0}^{M} \lambda _k \varPhi _k \, , \, w \, \right\rangle \quad \text {s.t.} \quad \Vert w \Vert _H \, \le \, \overline{D}_C(M) \; , \end{aligned}$$

where $\lambda \in \mathbb R^{M+1}$ are multipliers associated with the constraints $\langle \varPhi _k , w \rangle = 0$ for $k \in \{ 0, \ldots , M \}$. Applying the Cauchy-Schwarz inequality gives

$$\begin{aligned} \forall \lambda \in \mathbb R^{M+1}, \quad \overline{V}_M \, \le \, \left\| g - \sum _{k=0}^{M} \lambda _k \varPhi _k \right\| _H \, \overline{D}_C(M)\,, \end{aligned}$$

and with the particular choice $\lambda _k^* := \frac{\langle g , \varPhi _k \rangle }{\sigma _k}$ for each $k\in \{0,\ldots ,M\}$, we have

$$\begin{aligned} \overline{V}_M \, \le \, \left\| g - P_M(g) \right\| _H \, \overline{D}_C(M) \, = \, R_C(M,g) \; . \end{aligned}$$

The optimal value of the minimization problem

$$\begin{aligned} \underline{V}_M \, := \, \inf _{x \in C} \; \langle g, x - P_M(x) \rangle \end{aligned}$$

can be estimated analogously, giving $\underline{V}_M\ge -R_C(M,g)$, and the result follows. $\square$

The following example establishes the regularity of piecewise smooth functions with a finite number of singularities in the Hilbert space of square-integrable functions with the Legendre polynomials as orthogonal basis functions.

### Example 2

We consider the Hilbert space $H = L_2[0,1]$ of standard square-integrable functions on the interval [0, 1] equipped with the standard inner product, $\langle f,g\rangle :=\int _0^1 f(s)g(s)\mathrm{d}s$, and we choose the Legendre polynomials on the interval [0, 1] with weighting factors $\sigma _k = \frac{1}{2k+1}$ as orthogonal basis functions $(\varPhi _k)_{k \in \mathbb N}$. Our focus is on piecewise smooth functions $g: [0,1] \rightarrow \mathbb R$ with a given finite number of singularities, for which we want to establish regularity in the sense of Definition 3 for a bounded constraint set $C\subset L_2[0,1]$.

There are numerous results on approximating functions using polynomials, including convergence rate estimates [15]. One such result in [48] shows that any piecewise smooth function $f: [0,1] \rightarrow \mathbb R$ can be approximated with a polynomial $p_f^M: [0,1] \rightarrow \mathbb R$ of degree *M* such that

5 $$\begin{aligned} \forall y\in [0,1], \quad \left\| f(y) - p_f^M(y) \right\| \le K_1 \exp \left( -K_2 M^\alpha d(y)^\beta \right) \,, \end{aligned}$$

for any given $\alpha ,\beta > 0$ with either $\alpha < 1$ and $\beta \ge \alpha$, or $\alpha = 1$ and $\beta > 1$; some constants $K_1,K_2 > 0$; and where *d*(*y*) denotes the distance to the nearest singularity. In particular, the following convergence rate estimate can be derived using this result in the present example, for any piecewise smooth functions $g: [0,1] \rightarrow \mathbb R$ with a finite number of singularities:

$$\begin{aligned} R_C(M&,g) \; = \; \left\| g - P_M(g) \right\| _2 \, \overline{D}_C(M) \; = \; \inf _{\lambda } \, \left\| \, g - \sum _{k=0}^{M} \lambda _k \varPhi _k \, \right\| _2 \, \overline{D}_C(M) \nonumber \\&\; \overset{\text {(Lemma 1)}}{\le } \inf _{\lambda } \, \left\| \, g - \sum _{k=0}^{M} \lambda _k \varPhi _k \, \right\| _2 \gamma \; \le \; \frac{K}{\sqrt{M}} \end{aligned}$$

for some constant $K < \infty$. In order to establish the very last part of the above inequality, it is enough to consider a function *g* with a single singularity, e.g., at the mid-point $y = \frac{1}{2}$, and using $\alpha = \beta = \frac{1}{2}$:^1^

6 $$\begin{aligned} \inf _{\lambda } \, \left\| \, g - \sum _{k=0}^{M} \lambda _k \varPhi _k \, \right\| _2\le & {} \sqrt{\int _0^1 \, K_1^2 \exp \left( -2 K_2 \sqrt{ M \left| y-1/2 \right| } \right) \, \mathrm {d}y} \nonumber \\= & {} \sqrt{\frac{K_1^2}{\left[ K_2 \sqrt{M} \right] ^2} + \mathbf {O}\left( \frac{1}{\sqrt{M}} \exp \left( - K_2 \sqrt{M}\right) \right) }\nonumber \\= & {} \mathbf {O}\left( M^{-1/2} \right) \; . \end{aligned}$$

Convergence rate estimates for *k*-times differentiable and piecewise smooth functions can be obtained in a similar way, using for instance the results in [15, 48]. $\square$

A useful generalization of Definition 3 and a corollary of Theorem 1 are given below.

### Definition 4

A set $G\subseteq H$ is said to be *regular for C* if

$$\begin{aligned} \lim _{M \rightarrow \infty } \, \overline{R}_C(M,G) \; = \; 0\quad \text {with}\quad \overline{R}_C(M,G) \; := \; \sup _{g \in G} \; R_C(M,g) \;. \end{aligned}$$

Moreover, we call the function $\overline{R}_C(\cdot ,G):\mathbb N\rightarrow \mathbb R^+$ the *worst-case convergence rate for G on C*.

### Corollary 1

For any regular set $G \subseteq H$, we have

$$\begin{aligned} \lim _{M \rightarrow \infty } \; \sup _{\begin{array}{c} g \in G, \\ x \in C \end{array}} \; \left| \, \langle g , x-P_M(x) \rangle \, \right| \; = \; 0 \; . \end{aligned}$$

### Remark 2

While any subset of the Euclidean space $\mathbb R^n$ is trivially regular for a given bounded subset $C\subset \mathbb R^n$, only certain subsets/subspaces of an infinite-dimensional Hilbert space happen to be regular. Consider for instance the space of square-integrable functions, $H:= L_2[a,b]$, and let $G^p$ be any subset of *p*-times differentiable functions on [*a*, *b*], with uniformly Lipschitz-continuous *p*-th derivatives. It can be shown—e.g., from the analysis in [27] using the standard trigonometric Fourier basis, or from the results in [55] using the Legendre polynomial basis—that

$$\begin{aligned} \overline{R}_C(M,G^p) \; \le \; \mathbf {O}\left( \log (M)M^{-p-1} \right) \; \le \; \mathbf {O}\left( M^{-p} \right) \,, \end{aligned}$$

for any bounded constraint set $C\subset L_2[a,b]$, and $G^p$ is thereby regular for *C*. This leads to a rather typical situation, whereby the stronger the regularity assumptions on the function class, the faster the convergence of the associated worst-case convergence rate $R(\cdot ,G^p)$—an increase in the convergence rate order $\log (M)M^{-p-1}$ with *p* in this instance. In the limit of smooth ($\mathscr {C}^\infty$) functions, it can even be established—e.g., using standard results from Fourier analysis [19, 28]—that the convergence rate becomes exponential,

$$\begin{aligned} \overline{R}_C(M,G^\infty ) \; \le \; \mathbf {O}\left( \exp (-\beta M)\right) \quad \text {with}\ \ \beta > 0\,. \end{aligned}$$

### Example 2

(*Continued*) Consider the following set of unit-step functions

$$\begin{aligned} G_t \; := \; \left\{ \, x_t \, | \, t \in [0,1] \right\} \quad \text {with} \quad \forall \tau \in [0,1], \ \ x_t(\tau ) := \left\{ \begin{array}{ll} 1 &{} \quad \text {if }\tau \le t,\\ 0 &{} \quad \text {otherwise,} \end{array} \right. \end{aligned}$$

for which we want to establish regularity in the sense of Definition 4. Using earlier results in Example 2, it is known that the function $x_{0.5}$ can be approximated with a sequence of polynomials $p_{0.5}^M: [0,1] \rightarrow \mathbb R$ of degree *M* such that

$$\begin{aligned} \left\| x_{0.5} - p_{0.5}^M \right\| _{2} \; \le \; \mathbf {O}\left( M^{-1/2} \right) \;. \end{aligned}$$

For every $t \in [0,1]$ likewise, we can construct the family of polynomials

$$\begin{aligned} \forall \tau \in [0,1], \quad p_t^M(\tau ) \; := \; p_{0.5}^M \left( \frac{1-t+\tau }{2} \right) \;. \end{aligned}$$

Since the latter satisfy the same property as $x_{0.5}$ that

$$\begin{aligned} \left\| x_{t} - p_{t}^M \right\| _{2} \; \le \; \frac{K}{\sqrt{M}}\;, \end{aligned}$$

where the constant $K < \infty$ is independent of *t* or *M*, we have $\overline{R}_C(M,G_t) \le \mathbf {O}\left( M^{-1/2} \right)$.

This example can be generalized to other classes of functions. For instance, given any smooth function $f \in L_2[0,1]$, the subset

$$\begin{aligned} G_f \; := \; \left\{ \, g \in H \, | \, \exists t \in [0,1]: \; g(\tau ) = f(\tau ) \; \text {if }\tau \le t; g(\tau ) = 0 \; \text {otherwise} \right\} \end{aligned}$$

is regular in *H*, and also satisfies $\overline{R}_C(M,G_f) \le \mathbf {O}\left( M^{-1/2}\right)$. This result can be established by writing the elements in $G_f$ as the product between the piecewise smooth function *f* and the function $x_t$, and then approximating the factors separately. $\square$

In the remainder of this section, we analyze and illustrate a regularity condition for the cost functional in Problem (1).

### Definition 5

The functional $F: H \rightarrow \mathbb R$ is said to be *strongly Lipschitz-continuous on C* if there exists a bounded subset $G \subset H$ which is regular on *C* and a constant $L < \infty$ such that

7 $$\begin{aligned} \forall e \in H, \quad \sup _{x \in C} \, \left| F(x+e) - F(x) \right| \, \le \, L \, \sup _{ g \in G } \, \left| \, \langle g , e \rangle \right| \; . \end{aligned}$$

### Remark 3

In the special case of an affine functional *F*, given by

$$\begin{aligned} F(x) \; := \; F_0 + \langle \hat{g} , x \rangle \end{aligned}$$

where $F_0 \in H$, and $\hat{g}\in H$ is a regular element for *C*, the condition (7) is trivially satisfied with $L = 1$ and $G = \{ \hat{g} \}$. In this interpretation, the regularity condition (7) essentially provides a means of keeping the nonlinear part of *F* under control.

### Remark 4

Consider the finite-dimensional Euclidean space $\mathbb R^{n}$, a bounded subset $S\subset \mathbb {R}^n$, and a continuously-differentiable function $F: \mathbb R^n \rightarrow \mathbb R$ whose first derivative is bounded in the subset $G \subset \mathbb R^n$. By the mean-value theorem, *F* satisfies

$$\begin{aligned} \forall e \in \mathbb R^n, \quad \sup _{x \in S} \, \left| F(x+e) - F(x) \right| \,=&\sup _{x \in S} \, \left| \int _0^1 \, \left\langle \frac{\partial F}{\partial x}(x+\eta e) , e \right\rangle \, \mathrm {d}\eta \right| \\ \le&\sup _{g \in G} \, \left| \, \langle g , e \rangle \, \right| \, . \end{aligned}$$

Thus, any continuously differentiable function with a bounded first derivative is strongly Lipschitz-continuous on any bounded subset of $\mathbb R^n$. This result can be generalized to certain classes of functionals in infinite-dimensional Hilbert space. For instance, let $F: H \rightarrow \mathbb R$ be Fréchet differentiable, such that

$$\begin{aligned} \forall (x,e) \in C\times H, \quad F(x+e) - F(x) \; = \; \int _0^1 \, \langle DF(x+\eta e) , e \rangle \, \mathrm {d}\eta \,, \end{aligned}$$

and let the set of Fréchet derivatives $G := \{ DF(x)\,\mid \, x\in H \}\subseteq H$ be both bounded and regular on *C*. Then, *F* is strongly Lipschitz-continuous on *C*.

The following two examples investigate strong Lipschitz continuity for certain classes of functionals in the practical space of square-integrable functions with the Legendre polynomials as orthogonal basis functions. The first one (Example 3) illustrates the case of a functional that is not strongly Lipschitz-continuous; the second one (Example 4) identifies a broad class of strongly Lipschitz-continuous functionals defined via the solution of an embedded ODE system. The intention here is to help the reader develop an intuition that strongly Lipschitz-continuous functionals occur naturally in many, although not all, problems of practical relevance.

### Example 3

We consider the Hilbert space $H = L_2[0,1]$ of square-integrable functions on the interval [0, 1] with the standard inner product, and select the orthogonal basis functions $(\varPhi _k)_{k \in \mathbb N}$ as the Legendre polynomials on the interval [0, 1] with weighting factors $\sigma _k = \frac{1}{2k+1}$. We investigate whether the functional *F* given below is strongly Lipschitz-continuous on the set $C := \left\{ x \in L_2[0,1] \,\mid \, \forall s\in [0,1],\; |x(s)| \le 1 \right\}$,

$$\begin{aligned} \forall x \in L_2[0,1], \quad F(x) \; := \; \Vert x \Vert _2^2 \; = \; \int _0^1 \, x(s)^2 \, \mathrm {d}s\,. \end{aligned}$$

Consider the family of sets defined by

$$\begin{aligned} \forall M \in \mathbb N, \quad E_M \; := \; \left\{ P_M(x)-x \mid \, x \in C \, \right\} \; \subseteq \; L_2[0,1]\,. \end{aligned}$$

If the condition (7) were to hold for some bounded and regular set *G*, we would have by Theorem 1 that

$$\begin{aligned} \sup _{\begin{array}{c} e \in E_M,\\ x \in C \end{array}} \left| F(x+e) - F(x) \right| \;\le \; L \, \sup _{\begin{array}{c} e \in E_M,\\ g \in G \end{array}} \left| \, \langle g , e \rangle \right| \;=\; L \, \sup _{\begin{array}{c} x \in C,\\ g \in G \end{array}} \left| \, \langle g , x-P_M(x) \rangle \right| \,, \end{aligned}$$

and it would follow from Corollary 1 that

$$\begin{aligned} \lim _{M \rightarrow \infty } \sup _{\begin{array}{c} e \in E_M,\\ x \in C \end{array}} \left| F(x+e) - F(x) \right| \;=\; 0\,. \end{aligned}$$

However, this leads to a contradiction since we also have

$$\begin{aligned} \forall M \in \mathbb N, \quad \sup _{\begin{array}{c} e \in E_M,\\ x \in C \end{array}} \left| F(x+e) - F(x) \right| \;\ge \; \sup _{e \in E_M} F(e) \;=\; \sup _{x \in C} \Vert x - P_M(x) \Vert _2^2 \;=\; 1 \,. \end{aligned}$$

Therefore, the regularity condition (7) may not be satisfied for any bounded and regular set *G*, and *F* is not strongly Lipschitz-continuous on *C*. $\square$

### Remark 5

The result that the functional *F* in Example 3 is not strongly Lipschitz-continuous on *C* is not in contradiction with Remark 4. Although *F* is Fréchet differentiable in $L_2[0,1]$, the corresponding set *G* of the Fréchet derivatives of *F* is indeed unbounded.

### Example 4

We again consider the Hilbert space $H = L_2[0,1]$ of square-integrable functions on the interval [0, 1] equipped with the standard inner product, and select the orthogonal basis functions $(\varPhi _k)_{k \in \mathbb N}$ as the Legendre polynomials on the interval [0, 1] with weighting factors $\sigma _k = \frac{1}{2k+1}$. Our focus is on the ordinary differential equation (ODE)

8 $$\begin{aligned} \forall t \in [0,1], \quad \frac{\partial x}{\partial t}(t,u) = f(x(t,u)) + B u(t) \quad \text {with} \quad x(0,u) = 0 \; , \end{aligned}$$

where $B \in \mathbb R^{n\times n}$ is a constant matrix; and $f: \mathbb R^n \rightarrow \mathbb R^n$, a continuously-differentiable and globally Lipschitz-continuous function, so that the solution trajectory $x(\cdot ,u): [0,1] \rightarrow \mathbb R^n$ is well-defined for all $u \in L_2[0,1]$. For simplicity, we consider the functional *F* given by

$$\begin{aligned} F(u) \; := \; c^\mathsf {T}x(1,u) \;, \end{aligned}$$

for some real vector $c \in \mathbb R^n$. Moreover, the constraint set $C \subseteq H$ may be any uniformly bounded function subset, such as simple uniform bounds of the form

$$\begin{aligned} C \,:=\, \left\{ u \in L_2[0,1] \,\mid \, \forall \tau \in [0,1],\; |u(\tau )| \le 1 \right\} \,. \end{aligned}$$

The following developments aim to establish that *F* is strongly Lipschitz-continuous on *C*.

By Taylor’s theorem, the defect $\delta (t,u,e) := x(t,u+e)-x(t,u)$ satisfies the differential equation

$$\begin{aligned} \forall t \in [0,1] , \quad \frac{\partial \delta }{\partial t}(t,u,e) \; = \; \varLambda (t,u,e) \delta (t,u,e) + B e(t) \end{aligned}$$

with $\delta (0,u,e)=0$ and $\varLambda (t,u,e) := \int _0^1 \, \frac{\partial f}{\partial x}( x(t,u) + \eta \delta (t,u,e) ) \, \mathrm {d}\eta$. The right-hand-side function *f* being globally Lipschitz-continuous, we have for any given smooth matrix-valued function $A: [0,1] \rightarrow \mathbb R^{n \times n}$,

$$\begin{aligned} \forall (t,u,e)\in [0,1]\times C\times H, \quad \Vert \varLambda (t,u,e) - A(t) \Vert \le \ell _1\,, \end{aligned}$$

for some constant $\ell _1 < \infty$. For a particular choice of *A*, we can decompose $\delta (t,u,e)$ into the sum $\delta _\mathrm{l}(t,e) + \delta _\mathrm{n}(t,u,e,\delta _\mathrm{l})$ corresponding to the solution of the ODE system

9 $$\begin{aligned} \forall t \in [0,1], \qquad \delta _\mathrm{l}(t,e)= & {} A(t) \delta _\mathrm{l}(t,e) + B e(t) \end{aligned}$$

10 $$\begin{aligned} \delta _\mathrm{n}(t,u,e,\delta _\mathrm{l})= & {} \varLambda (t,u,e) \delta _\mathrm{n}(t,u,e,\delta _\mathrm{l}) + [\varLambda (t,u,e)-A(t)]\delta _\mathrm{l}(t,e) \nonumber \\ \end{aligned}$$

with $\delta _\mathrm{l}(0,e) = \delta _\mathrm{n}(0,u,e,\delta _\mathrm{l}) = 0$. In this decomposition, the left-hand side of (7) satisfies

$$\begin{aligned} \forall e \in H, \quad \sup _{u \in C} \, \left| F(u+e) - F(u) \right| \, \le \, \left| c^\mathsf {T}\delta _\mathrm{l}(1,e) \right| + \sup _{u \in C} \left| c^\mathsf {T}\delta _\mathrm{n}(1,u,e) \right| \,. \end{aligned}$$

Regarding the linear term $\delta _\mathrm{l}$ first, we have

11 $$\begin{aligned} \forall s \in [0,1], \quad c^\mathsf {T}\delta _\mathrm{l}(s,e) = \langle g_s, e \rangle \end{aligned}$$

with

$$\begin{aligned} \forall t \in [0,1], \quad g_s(t) := \left\{ \begin{array}{ll} \displaystyle \int _0^t c^\mathsf {T}\varGamma (t,\tau ) B \, \mathrm {d}\tau &{} \text { if }t \le s, \\ 0 &{} \text { otherwise,} \end{array} \right. \end{aligned}$$

where $\varGamma (t,\tau )$ denotes the fundamental solution of the linear ODE (9) such that

$$\begin{aligned} \forall (\tau ,t) \in [0,1]^2, \quad \frac{\partial }{\partial t} \varGamma (t,\tau ) \; = \; A(t)\varGamma (t,\tau ) \quad \text {with} \quad G(\tau ,\tau ) = I \; . \end{aligned}$$

Since *A* is smooth, it follows from Example 2 that the set $G:= \{ g_s \mid s \in [0,1] \}$ is both regular on *C* and bounded, and satisfies

$$\begin{aligned} \overline{R}_C(M,G) \; \le \; \mathbf {O}\left( M^{1/2}\right) \,. \end{aligned}$$

Regarding the nonlinear term $\delta _\mathrm{n}$, since the function $\varLambda$ is uniformly bounded, applying Gronwall’s lemma to the ODE (10) gives

12 $$\begin{aligned} \forall (t,u,e)\in [0,1]\times C\times H, \quad c^\mathsf {T}\delta _\mathrm{n}(t,u,e,\delta _\mathrm{l}) \; \le \;&\ell \exp (\ell ) \, \sup _{s \in [0,1]} \, | c^\mathsf {T}\delta _\mathrm{l}(s,e)| \nonumber \\ \le \;&\ell \exp (\ell ) \, \sup _{g \in G} \, | \langle g, e \rangle | \,, \end{aligned}$$

for some constant $\ell <\infty$. Finally, combining (11) and (12) shows that *F* satisfies the condition (7) with $L := 1 + \ell \exp (\ell )$, thus *F* is strongly Lipschitz-continuous on *C*. $\square$

### Remark 6

The functional *F* in the previous example is defined implicitly via the solution of an ODE. The result that such functionals are strongly Lipschitz-continuous is particularly significant insofar as the proposed optimization framework will indeed encompass a broad class of optimal control problems as well as problems in the calculus of variations. In fact, it turns out that strong Lipschitzness still holds in replacing the constant matrix *B* in (8) with any matrix-valued continuously differentiable and globally Lipschitz-continuous function of *x*(*t*, *u*), thus encompassing quite a general class of nonlinear affine-control systems. In the case of general nonlinear ODEs, however, strong Lipschitzness may be lost. Strong Lipschitzness could nevertheless be recovered by restricting condition (7) in Definition 5 as

$$\begin{aligned} \forall e \in E_C, \quad \sup _{x \in C} \, \left| F(x+e) - F(x) \right| \, \le \, L \, \sup _{ g \in G } \, \left| \, \langle g , e \rangle \right| \; , \end{aligned}$$

with the projection error set $E_C \; := \; \left\{ P_M(x)-x \, | \, x \in C, \, M \in \mathbb N \, \right\} \subset H$, and also restricting the constraint set *C* to only contain uniformly bounded and Lipschitz-continuous functions in $L_2[0,1]$ with uniformly bounded Lipschitz constants.

We close this section with a brief analysis of the relationship between strong and classical Lipschitzness in infinite-dimensional Hilbert space.

### Lemma 2

Every strongly Lipschitz-continuous functional $F:H\rightarrow \mathbb R$ on *C* is also Lipschitz-continuous on *C*.

### Proof

Let *G* be a bounded and regular subset of *H* on *C* such that the condition (7) is satisfied. Since *G* is bounded, there exists a constant constant $\alpha <\infty$ such that $\sup _{g \in G} \left\| g \right\| _H \le \alpha$. Applying the Cauchy–Schwarz inequality to the right-hand side of (7) gives

$$\begin{aligned} \forall e \in H, \quad \sup _{x \in C} \, \left| F(x+e) - F(x) \right| \, \le \, L \, \alpha \, \left\| e \right\| _H \; , \end{aligned}$$

and so *F* is Lipschitz-continuous on *C*. $\square$

### Remark 7

With regularity of the set *G* alone, i.e. without boundedness, the condition (7) may not imply Lipschitz-continuity, or even continuity of *F*. As a counter-example, let $G := \mathrm {span} \left( \varPhi _0, \varPhi _1, \ldots , \varPhi _N \right)$ be the subspace spanned by the first *N* basis functions in the infinite-dimensional Hilbert space *H*. It is clear that *G* is regular on any bounded set $C\subset H$ since $\overline{R}_C(M,G) = 0$ for all $M \ge N$. Now, let the functional $F:H\rightarrow \mathbb R$ given by

$$\begin{aligned} F(x) := \left\{ \begin{array}{ll} 0 &{} \quad \text {if} \; \langle \hat{g} , x \rangle \le 0 \\ 1 &{} \quad \text {otherwise} \end{array} \right. \end{aligned}$$

for some $\hat{g} \in G$. For every $(x,e) \in C\times H$, we have

$$\begin{aligned} \left| F(x+e) - F(x) \right| \;\le \; \left\{ \begin{array}{ll} 0 &{} \text {if} \; \langle \hat{g} , e \rangle = 0 \\ 1 &{} \text {otherwise} \end{array} \right\} \;\le \; \left\{ \begin{array}{ll} 0 &{} \text {if} \; P_N(e) = 0 \\ \infty &{} \text {otherwise} \end{array} \right\} \;=\; \sup _{ g \in G } \, \left| \, \langle g , e \rangle \right| \; . \end{aligned}$$

Therefore, despite being discontinuous, the condition (7) is indeed satisfied.

### Remark 8

In general, Lipschitz-continuity does not imply strong Lipschitz-continuity in an infinite-dimensional Hilbert space. A counter-example is easily contrived for the functional $F:L_2[0,1]\rightarrow \mathbb R$ given by

$$\begin{aligned} \forall x \in L_2[0,1], \quad F(x) \; := \; \max \{ 1, \Vert x \Vert _2^2 \} \; . \end{aligned}$$

Although this functional is Lipschitz-continuous, it can be shown by a similar argument as in Example 3 that it is not strongly Lipschitz-continuous.

## Global optimization in Hilbert space using complete search

The application of complete-search strategies to infinite-dimensional optimization problems such as (1) calls for an extension of the (spatial) branch-and-bound principle [23] to general Hilbert space. The approach presented in this section differs from branch-and-bound in that the dimension *M* of the search space is adjusted, as necessary, during the iterations of the algorithm, by using a so-called *lifting* operation—hence the name *branch-and-lift* algorithm. The basic idea is to bracket the optimal solution value of Problem (1) and progressively refine these bounds via this lifting mechanism, combined with traditional branching and fathoming.

Based on the developments in Sect. 2, the following subsections describe methods for exhaustive partitioning in infinite-dimensional Hilbert space (Sect. 3.1) and for computing rigorous upper and lower bounds on given subsets of the variable domain (Sect. 3.2), before presenting the proposed branch-and-lift algorithm (Sect. 3.3).

### Partitioning in infinite-dimensional Hilbert space

Similar to branch-and-bound search, the proposed branch-and-lift algorithm maintains a partition $\mathscr {A} := \{A_1,\ldots ,A_k\}$ of finite-dimensional sets $A_1,\ldots ,A_k$. This partition is updated through the repeated application of certain operations, including branching and lifting, in order to close the gap between an upper and a lower bound on the global solution value of the optimization problem (1). The following definition is useful in order to formalize these operations:

#### Definition 6

With each pair $(M,A) \in \mathbb N \times \mathscr {P}( \mathbb R^{M+1})$, we associate a subregion $X_M(A)$ of *H* given by

$$\begin{aligned} X_M(A) \; := \; \left\{ \, x \in C \, \left| \, \left( \frac{\langle x, \varPhi _0 \rangle }{\sigma _0}, \ldots , \frac{\langle x, \varPhi _M \rangle }{\sigma _M} \right) ^\mathsf {T}\, \in \, A \, \right. \right\} \, . \end{aligned}$$

Moreover, we say that the set *A* is *infeasible* if $X_M(A) = \varnothing$.

Notice that each subregion $X_M(A)$ is a convex set if the sets *C* and *A* are themselves convex. For practical reasons, we restrict ourselves to compact subsets $A \in \mathbb S^{M+1} \subseteq \mathscr {P}( \mathbb R^{M+1})$ herein, where the class of sets $\mathbb S^{M+1}$ is easily stored and manipulated by a computer. For example, $\mathbb S^{M+1}$ could be a set of interval boxes, polytopes, ellipsoids, etc.

The ability to detect infeasibility of a set $A \in \mathbb S^{M+1}$ is pivotal for complete search. Under the assumption that the constraint set *C* is convex (Assumption 1), a certificate of infeasibility can be obtained by considering the convex optimization problem

13 $$\begin{aligned} d_C(A) \; := \; \min _{x,y \in H} \Vert x - y \Vert _H \quad \text {s.t.} \quad \left( \frac{\langle y, \varPhi _0 \rangle }{\sigma _0}, \ldots , \frac{\langle y, \varPhi _M \rangle }{\sigma _M} \right) ^\mathsf {T}\, \in \, A \; , \; \; x \in C.\nonumber \\ \end{aligned}$$

It readily follows from the Cauchy–Schwarz inequality that

$$\begin{aligned} -\Vert x - y \Vert _H \le \langle x, \varPhi _k \rangle - \langle y, \varPhi _k \rangle \le \Vert x - y \Vert _H\;, \end{aligned}$$

for any (normalized) basis function $\varPhi _k$, and so $\Vert x-y \Vert _H = 0$ implies $\langle x, \varPhi _k \rangle = \langle y, \varPhi _k \rangle$. Consequently, a set *A* is infeasible if and only if $d_C(A) > 0$. Because Slater’s constraint qualification holds for Problem (13) under Assumption 1, one approach to checking infeasibility to within high numerical accuracy relies on duality for computing lower bounds on the optimal solution value $d_C(A)$—similar in essence to the infinite-dimensional convex optimization techniques in [4, 14]. For the purpose of this paper, our focus is on a general class of non-convex objective functionals *F*, whereas the constraint set *C* is assumed to be convex and to have a simple geometry in order to avoid numerical issues in solving feasibility problems of the form (13). We shall therefore assume, from this point onwards, that infeasibility can be verified with high numerical accuracy for any set $A \in \mathbb S^{M+1}$.

A *branching* operation subdivides any set $A \in \mathbb S^{M+1}$ in the partition $\mathscr {A}$ into two compact subsets $A_\mathrm{l},A_\mathrm{r} \in \mathbb S^{M+1}$ such that $A_\mathrm{l}\cup A_\mathrm{r} \supseteq A$, thereby updating the partition as

$$\begin{aligned} \mathscr {A} \,\leftarrow \mathscr {A} \setminus \{A\} \cup \{A_\mathrm{l}, A_\mathrm{r}\}\,. \end{aligned}$$

On the other hand, a *lifting* operation essentially lifts any set $A \in \mathbb S^{M+1}$ into a higher-dimensional space under the function $\varGamma _M: \mathbb S^{M+1} \rightarrow \mathbb S^{M+2}$, defined such that

$$\begin{aligned} \forall A \in \mathbb S^{M+1}, \quad X_M(A) \; \subseteq \; X_{M+1}( \varGamma _M(A) )\,. \end{aligned}$$

The question as to defining the higher-order coefficient $\langle x, \varPhi _{M+1} \rangle$ in such a lifting is related to the so called *moment problem* that asks the question under which conditions on a sequence $(a_k)_{k \in \{ 1,\ldots ,N\}}$, named moment sequence, can we find an associated element $x \in H$ with $a_ k = \frac{\langle x, \varPhi _k \rangle }{\sigma _k}$ for each $k \in \{ 1, \ldots , N \}$. Classical examples of such moment problems are Stieltjes’, Hamburger’s, and Legendre’s moment problems [1]. Here, we adopt the modern standpoint on moment problems using convex optimization [30, 42], by considering the following optimization subproblems:

14 $$\begin{aligned} {\underline{a}}_{M+1}(A) \;\le \; \min _{x \in X_M(A)} \, \frac{ \langle x, \varPhi _{M+1} \rangle }{\sigma _{M+1}} \;\quad \text {and} \;\quad {\overline{a}}_{M+1}(A) \;\ge \; \max _{x \in X_M(A)} \, \frac{ \langle x, \varPhi _{M+1} \rangle }{\sigma _{M+1}}.\nonumber \\ \end{aligned}$$

Although both optimization problems in (14) are convex when *A* and *C* are convex, they remain infinite-dimensional, and thus intractable in general. Obtaining lower and upper bounds $\underline{a}_{M+1}(A)$, $\overline{a}_{M+1}(A)$ is nonetheless straightforward under Assumption 1. In case no better approach is available, one can always use

$$\begin{aligned} {\underline{a}}_{M+1}(A) := - \frac{\gamma }{\sigma _{M+1}} \quad \text {and} \quad {\overline{a}}_{M+1}(A) := \frac{\gamma }{\sigma _{M+1}} \; , \end{aligned}$$

which follows readily from the Cauchy–Schwarz inequality and the property that $\Vert \varPhi _{M+1} \Vert _H = 1$. As already mentioned in the introduction of the paper, a variety of algorithms are now available for tackling convex infinite dimensional problems both efficiently and reliably [4, 14], which could provide tighter bounds in practical applications.

A number of remarks are in order:

#### Remark 9

The idea to introduce a lifting operation to enable partition in infinite-dimensional function space was originally introduced by the authors in a recently publication [25], focusing on global optimization of optimal control problems. One principal contribution in the present paper is a generalization of these ideas to global optimization in any Hilbert space, by identifying a set of sufficient regularity conditions on the cost functional and constraint set for the resulting branch-and-lift algorithms to converge to an $\varepsilon$-global solution in finite run-time.

#### Remark 10

Many recent optimization techniques for global optimization are based on the theory of positive polynomials and their associated linear matrix inequality (LMI) approximations [30, 45], which are also originally inspired by moment problems. Although these LMI techniques may be applied in the practical implementation of the aforementioned lifting operation, they are not directly related to the branch-and-lift algorithm that is developed in the following sections. An important motivation for moving away from the generic LMI framework is that the available implementations scale quite poorly with the number of optimization variables, due to the combinatorial increase of the number of monomials in the associated multivariate polynomial. Therefore, a direct approximation of the cost function *F* with multivariate polynomials would conflict with our primary objective to develop a global optimization algorithm whose worst-case run-time does not depend on the number of optimization variables.

### Strategies for upper and lower bounding of functionals

Besides partitioning, the efficient construction of tight upper and lower bounds on the global solution value of (1) for given subregions of *H* is key in a practical implementation of branch-and-lift. Thereafter, functions $L_M,U_M: \mathbb S^{M+1} \rightarrow \mathbb R$ such that

15 $$\begin{aligned} \forall A \in \mathbb S^{M+1}, \quad L_M(A) \; \le \; \inf _{x \in X_M(A)} \, F(x) \, \le \, U_M(A) \, , \end{aligned}$$

shall be call lower- and upper-bounding functions of the functional *F*, respectively. A simple approach to constructing these lower and upper bounds relies on the following two-step decomposition:

1. Compute bounds $L^0_M(A)$ and $U^0_M(A)$ on the finite-dimensional approximation of *F* as
  16 $$\begin{aligned} \forall A \in \mathbb S^{M+1}, \quad L^0_M(A) \; \le \; \inf _{a \in A} \, F \left( \sum _{i=0}^M a_i \varPhi _i \right) \; \le \; U^0_M(A) \, . \end{aligned}$$
  Clearly, it depends on the particular expression of *F* how to determine such bounds in practice. In the case that *F* is factorable, various arithmetics can be used to propagate bounds through a DAG of the function, including interval arithmetic [36], McCormick relaxations [9, 33], and Taylor/Chebyshev model arithmetic [10, 43, 47]. Moreover, if the expression of *F* is embedding a dynamic system described by differential equations, validated bounds can be obtained by using a variety of set-propagation techniques as described, e.g., in [26, 31, 38, 50, 53]; or via hierarchies of LMI relaxations as in [21, 29].
2. Compute a bound $\varDelta _M(A)$ on the approximation errors such that
  17 $$\begin{aligned} \forall A \in \mathbb S^{M+1}, \quad&\left| \inf _{x \in X_M(A)} \, F(x) - \inf _{a \in A} \, F \left( \sum _{i=0}^M a_i \varPhi _i \right) \right| \;\le \; \varDelta _M(A) \, . \end{aligned}$$
  In the case that *F* is strongly Lipschitz-continuous on *C*, we can always take $\varDelta _M(A) := L \, \overline{R}_C(M,G)$, where the constant $L<\infty$ and the bounded regular set *G* satisfy the condition (7). Naturally, better bounds may be derived by exploiting a particular structure or expression of *F*.

By construction, the lower-bounding function $L_M(A):=L^0_M(A)-\varDelta _M(A)$ and the upper-bounding function $U_M(A):=U^0_M(A)+\varDelta _M(A)$ trivially satisfy (15). Moreover, when the set $A \in \mathbb S^{M+1}$ is infeasible—see related discussion in Sect. 3.1—we may set $\varDelta _M(A) = L_M(A) = U_M(A) = \infty$.

We state the following assumptions in anticipation of the convergence analysis in Sect. 4.

#### Assumption 3

The cost functional *F* in Problem (1) is strongly Lipschitz-continuous on *C*, with the condition (7) holding for the constant $L<\infty$ and the bounded regular subset $G\subset H$.

#### Remark 11

Under Assumption 3, Lemma 2 implies that

$$\begin{aligned} \forall a,a'\in A, \quad \left| F \left( \sum _{k=0}^M a_k \varPhi _k \right) - F \left( \sum _{k=0}^M a'_k \varPhi _k \right) \right| \,\le \, L' \, \left\| \sum _{k=0}^M (a_k-a'_k) \varPhi _k \right\| _H \end{aligned}$$

for a Lipschitz constant $L' \ge L \, \sup _{g \in G} \Vert g \Vert _H$. Thus, if Assumption 2 is also satisfied, any pair $(M,A) \in \mathbb N \times \mathbb S^{M+1}$ is such that

$$\begin{aligned}&\forall a,a'\in A, \quad \left| F \left( \sum _{k=0}^M a_k \varPhi _k \right) - F \left( \sum _{k=0}^M a'_k \varPhi _k \right) \right| \\&\quad \le L' \, \sum _{k=0}^M |a_k-a'_k| \left\| \varPhi _k \right\| \,\le \, K \, d_1(A) \end{aligned}$$

with $K := L \, \sup _{k\in \mathbb N} \left\| \varPhi _k \right\| _H$ and $d_1(A):=\sum _{i=0}^M \sup _{a,a'\in A}|a_i-a'_i|$. It follows that

$$\begin{aligned} \forall (M,A)\in \mathbb N\times \mathbb S^{M+1}, \quad U_M(A) - L_M(A) \; \le \; K\,d_1(A) + 2\,L \, \overline{R}_C(M,G)\,, \end{aligned}$$

and therefore the gap $U_M(A) - L_M(A)$ can be made arbitrarily small under Assumption 3 by choosing a sufficiently large order *M* and a sufficiently small diameter for the set *A*. This result will be exploited systematically in the convergence analysis in Sect. 4.

#### Remark 12

An alternative upper bound $U_M(A)$ in (15) may be computed more directly by solving the following nonconvex optimization problem to local optimality,

18 $$\begin{aligned} \min _{a \in A} \, F \left( \sum _{k=0}^M a_k \varPhi _k \right) \quad \mathrm {s.t.} \quad \sum _{k=0}^M a_k \varPhi _k \in C \,. \end{aligned}$$

Without further assumptions on the orthogonal basis functions $\varPhi _0, \varPhi _1, \ldots$ and on the constraint set *C*, however, it is not hard to contrive examples where $P_M(x) \notin C$ for all $x \in C$ and all $M \in \mathbb N$; that is, contrive examples where the upper bound (18) does not converge as $M \rightarrow \infty$. This upper-bounding approach could nonetheless be combined with another bounding approach based on set arithmetics in order to prevent convergence issues; e.g., use the solution value of (18) as long as it provides a bound that is smaller than $U^0_M(A)+\varDelta _M(A)$.

### Branch-and-lift algorithm

The foregoing considerations on partitioning and bounding in Hilbert space can be combined in Algorithm 1 for solving infinite-dimensional optimization problems to $\varepsilon$-global optimality. 
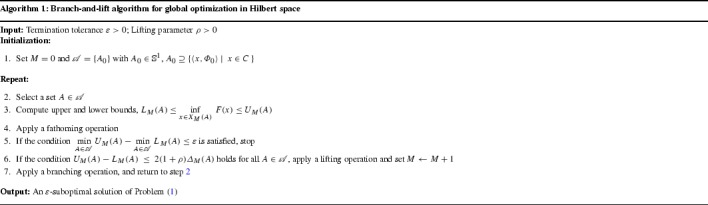


A number of remarks are in order:

- Regarding initialization, the branch-and-lift iterations starts with $M = 0$. A possible way of initializing the partition $\mathscr {A} = \left\{ A_0 \right\}$ is by noting that
  $$\begin{aligned} \{ \langle x, \varPhi _0 \rangle \mid \, x \in C \, \} \; \subseteq \; \left[ -\frac{\gamma }{\sigma _0}, \frac{\gamma }{\sigma _0}\right] \end{aligned}$$
  under Assumption 1.
- Besides the branching and lifting operations introduced earlier in Sect. 3.1, fathoming in Step 4 of Algorithm 1 refers to the process of discarding a given set $A\in \mathscr {A}$ from the partition if
  $$\begin{aligned} \quad L_M(A) \, = \, \infty \quad \text {or} \quad \exists \, A' \in \mathscr {A}: \; \; L_M(A) \, > \, U_{M}(A') \, . \end{aligned}$$
- The main idea behind the lifting condition defined in Step 6 of Algorithm 1, namely
  19 $$\begin{aligned} \forall A\in \mathscr {A}, \quad U_{M}(A)-L_{M}(A) \; \le \; 2(1+\rho ) \varDelta _M(A)\,, \end{aligned}$$
  is that a subset *A* should be lifted to a higher-dimensional space whenever the approximation error $\varDelta _M(A)$ due to the finite parameterization becomes of the same order of magnitude as the current optimality gap $U_{M}(A)-L_{M}(A)$. The aim here is to apply as few lifts as possible, since it is preferable to branch in a lower dimensional space. The convergence of the branch-and-lift algorithm under this lifting condition is examined in Sect. 4 below. Notice also that a lifting operation is applied globally—that is, to all parameter subsets in the partition $\mathscr {A}$–in Algorithm 1, so all the subsets in $\mathscr {A}$ share the same parameterization order at any iteration. In a variant of Algorithm 1, one could also imagine a family of subsets that would have different parameterization orders by applying the lifting condition locally instead.
- Finally, it will be established in the following section that, upon termination and under certain assumptions, Algorithm 1 returns an $\varepsilon$-suboptimal solution of Problem (1). In particular, Assumption 1 rules out the possibility of an infeasible solution.

## Convergence analysis of branch-and-lift

This section investigates the convergence properties of the branch-and-lift algorithm (Algorithm 1) developed previously. It is convenient to introduce the following notation in order to conduct the analysis:

### Definition 7

Let $G \subseteq H$ be a regular set for *C*, and define the inverse function $\overline{R}_C^{-1}(\cdot , G): \mathbb R^{++} \rightarrow \mathbb N$ by

$$\begin{aligned} \forall \varepsilon > 0, \quad \overline{R}_C^{-1}( \varepsilon , G ) \; := \; \min _{M \in \mathbb N} \; M \; \; \mathrm {s.t.} \; \; \overline{R}_C(M,G) \le \varepsilon \; . \end{aligned}$$

The following result is a direct consequence of the lifting condition (19) in the branch-and-lift algorithm:

### Lemma 3

Let Assumption 3 hold, and suppose that finite bounds $L^0_M(A)$, $U^0_M(A)$ and $\varDelta _M(A)$ satisfying (16)–(17) can be computed for any feasible pair $(M,A)\in \mathbb {N}\times \mathbb S^{M+1}$. Then, the number of lifting operations in a run of Algorithm 1 as applied to Problem (1) is at most

$$\begin{aligned} \overline{M} := \overline{R}_C^{-1}\left( \frac{\varepsilon }{2 (\rho +1)L} \, , \, G \right) \,, \end{aligned}$$

regardless of whether or not the algorithm terminates finitely.

### Proof

Assume that $M=\overline{M}$ in Algorithm 1, and that the termination condition is not yet satisfied; that is,

$$\begin{aligned} U_{\overline{M}}(A) - L_{\overline{M}}(A) > \varepsilon \end{aligned}$$

for a certain feasible set $A \in \mathscr {A}$. If the lifting condition (19) were to hold for *A*, then it would follow from (16)–(17) that

$$\begin{aligned} \varepsilon - 2\varDelta _{\overline{M}}(A) \; < \; U^0_{\overline{M}}(A)- L^0_{\overline{M}}(A) \; \le \; 2\rho \varDelta _{\overline{M}}(A) \, . \end{aligned}$$

Moreover, *F* being strongly Lipschitz-continuous on *C* by Assumption 3, we would have

$$\begin{aligned} \overline{R}_C({\overline{M}}, G) \; > \; \frac{\varepsilon }{2 (\rho +1)L} \; . \end{aligned}$$

This is a contradiction, since $\overline{R}_C({\overline{M}}, G) \le \frac{\varepsilon }{2 (\rho +1)L}$ by Definition 7. $\square$

Besides having a finite number of lifting operations, the convergence of Algorithm 1 can be established if the elements of a partition can be made arbitrarily small after applying a finite number of subdivisions.

### Definition 8

A partitioning scheme is said to be *exhaustive* if, given any dimension $M\in \mathbb N$, any tolerance $\eta >0$, and any bounded initial partition $\mathscr {A}=\{A_0\}$, we have

$$\begin{aligned} \mathrm {diam}\left( \mathscr {A}\right) \; := \; \max _{A \in \mathscr {A}} \; \mathrm {diam}\left( A\right) \; < \; \eta \,, \end{aligned}$$

after finitely many subdivisions, where $\mathrm {diam}\left( A\right) := \sup _{a,a' \in A} \; \Vert a - a' \Vert$. Moreover, we denoted by $\varSigma (\eta ,M)$ an upper bound on the corresponding number of subdivisions in an exhaustive scheme.

The following theorem provides the main convergence result for the proposed branch-and-lift algorithm.

### Theorem 2

Let Assumptions 1, 2 and 3 hold, and suppose that finite bounds $L^0_M(A)$, $U^0_M(A)$ and $\varDelta _M(A)$ satisfying (16)–(17) can be computed for any feasible pair $(M,A)\in \mathbb {N}\times \mathbb S^{M+1}$. If the partitioning scheme is exhaustive, then Algorithm 1 terminates after at most $\overline{\varSigma }$ iterations, where

20 $$\begin{aligned} {\overline{\varSigma }} \; \le \; \max _{0 \le M\le \overline{M}} \varSigma \left( \frac{\varepsilon \rho }{K (\rho +1)} , M \right) \,, \quad \text {with}~~K := L \, \sup _{k\in \mathbb N} \Vert \varPhi _k \Vert _H\,. \end{aligned}$$

### Proof

By Lemma 3, the maximal number *M* of lifting operations during a run of Algorithm 1 is finite, such that $M \le \overline{M}$. Therefore, the lifting condition (19) may not be satisfied for any feasible subset $A\in \mathscr {A}$, and we have

$$\begin{aligned} \varDelta _{M}(A) \,\le \, \frac{U^0_{M}(A)- L^0_{M}(A)}{2\,\rho } \,. \end{aligned}$$

Since $L_M(A)=L^0_M(A)-\varDelta _M(A)$ and $U_M(A)=U^0_M(A)+\varDelta _M(A)$, it follows that the termination condition $U_{M}(A)- L_{M}(A) \le \varepsilon$ is satisfied if

$$\begin{aligned} U^0_{M}(A)- L^0_{M}(A) \; \le \; \frac{\rho \varepsilon }{\rho +1} \; . \end{aligned}$$

By Assumptions 2 and 3 and Remark 11, we have

$$\begin{aligned} U^0_{M}(A)- L_{M}(A) \; \le \; K \, \mathrm {diam}\left( \mathscr {A}\right) \,, \end{aligned}$$

and the termination condition is thus satisfied if

$$\begin{aligned} \mathrm {diam}\left( \mathscr {A}\right) \;\le \; \frac{\varepsilon \rho }{K (\rho +1)}\,. \end{aligned}$$

This latter condition is met after at most $\varSigma \left( \frac{\varepsilon \rho }{K (\rho +1)} , M \right)$ iterations under the assumption that the partitioning scheme is exhaustive. $\square$

### Remark 13

In the case that the sets $A \in \mathscr {A}$ are simple interval boxes and the lifting process is implemented per (14), we have

$$\begin{aligned} \forall k\in \{0,\ldots ,M\}, \quad \left[ \, \underline{a}_{k}(A),\overline{a}_{k}(A) \, \right] \; \subseteq \; \left[ -\frac{\gamma }{\sigma _k}, \frac{\gamma }{\sigma _k} \right] \,. \end{aligned}$$

Therefore, one can always subdivide these boxes in such a way that the condition $\mathrm {diam}\left( \mathscr {A}\right) \le \eta$ is satisfied after at most $\varSigma (\eta ,M)$ subdivisions, with

$$\begin{aligned} \varSigma (\eta ,M) \; := \; \prod _{k=0}^M \left\lceil \frac{\gamma }{\eta \,\sigma _k} \right\rceil \; \in \; \mathbb N\,, \end{aligned}$$

for any given dimension *M*. In particular, $\varSigma (\eta ,M)$ is monotonically increasing in *M*, and (20) simplifies to

$$\begin{aligned} \overline{\varSigma }\; \le \; \varSigma \left( \frac{\varepsilon \rho }{K (\rho +1)} , \overline{M} \right) \,. \end{aligned}$$

It should be clear, at this point, that the worst-case estimate $\overline{\varSigma }$ given in Theorem 2 may be extremely conservative, and the performance of Algorithm 1 could be much better in practice. Nonetheless, a key property of this estimate $\overline{\varSigma }$ is that it is independent of the actual nature or the number of optimization variables in Problem (1), be it a finite-dimensional or even an infinite-dimensional optimization problem. As already pointed in the introduction of the paper, this result is quite remarkable since available run-time estimates for standard convex and non-convex optimization algorithms do not enjoy this property. On the other hand, $\overline{\varSigma }$ is dependent on:

- the bound $\gamma$ on the constraint set *C*;
- the Lipschitz constants *K* and *L* of the cost functional *F*;
- the uniform bound $\sup _k \Vert \varPhi _k \Vert _H$ and the scaling factors $\sigma _k$ of the chosen orthogonal functions $\varPhi _k$; and
- the lifting parameter $\rho$ and the termination tolerance $\varepsilon$ in Algorithm 1.

All these dependencies are illustrated in the following example.

### Example 5

Consider the space of square-integrable functions $H:=L_2[-\,\,\pi ,\pi ]$, for which it has been established in Remark 2 that any subset $G^p$ of *p*-times differentiable functions with uniformly Lipschitz-continuous *p*-th derivatives on $[-\,\,\pi ,\pi ]$ is regular, with convergence rate $\overline{R}_C(M,G^p) \le \alpha M^{-p}$ for some constant $\alpha <\infty$. On choosing the standard trigonometric Fourier basis, such that $\sigma _k=\pi$ are constant scaling factors and $K' := K\sup _k \Vert \varPhi _k \Vert _2 = K$, and doing the partitioning using simple interval boxes as in Remark 13, a worst-case iteration count can be obtained as

$$\begin{aligned} \overline{\varSigma }\; = \; \left( \, \left\lceil \frac{\gamma K (\rho +1)}{\pi \, \rho \, \varepsilon } \right\rceil \right) ^{\left\lceil \left( 2 \alpha (\rho +1)L/\varepsilon \right) ^\frac{1}{p} \right\rceil } \; \le \; \exp \left( \mathbf {O}\left( \left( 1/\varepsilon \right) ^{\frac{1}{p}} \, \log (1/\varepsilon ) \right) \right) \, . \end{aligned}$$

Furthermore, if the global minimizer of Problem (1) happens to be a smooth ($\mathscr {C}^\infty$) function, the convergence rate can be expected to be of the form $R(M,G^\infty ) = \alpha \exp (-\,\,\beta M)$, and Theorem 2 then predicts a worst-case iteration count as

$$\begin{aligned} \overline{\varSigma }\; \le \; \exp \left( \mathbf {O}\left( (\log (1/\varepsilon ))^2 \right) \right) \,, \end{aligned}$$

which is much more favorable. $\square$

## Numerical case study

We consider the Hilbert space $H := L_2[0,T]$ of square-integrable functions on the interval [0, *T*], here with $T=10$. Our focus is on the following nonconvex, infinite-dimensional optimization problem

21 $$\begin{aligned} \inf _{x\in L_2[0,T]} F(x)&:= \int _0^{T} \; \left[ \left( \int _0^T f_1(t-t') x(t') \, \mathrm {d}t' \right) ^2 - \left( \int _0^T f_2(t-t') x(t') \, \mathrm {d}t' \right) ^2 \right] \, \mathrm {d}t\nonumber \\ \mathrm{s.t.}\ \ x \in C&:= \left\{ \, x \in H \, \mid \forall t \in [0,T], \; |x(t)| \le 1 \right\} \,, \end{aligned}$$

with the functions $f_1$ and $f_2$ given by

$$\begin{aligned} \forall t \in \mathbb R, \quad f_1(t) = \frac{t}{2}\left( \sin \left( \frac{\pi t}{2T} \right) +1 \right) \quad \text {and} \quad f_2 = \frac{\partial f_1}{\partial t} \; . \end{aligned}$$

Notice the symmetry in the optimization problem (21), as $F(x) = F(-\,\,x)$ and $x \in C$ if and only if $-x \in C$. Thus, if $x^*$ is a global solution point of (21), then $-\,\,x^*$ is also a global solution point.

Although it might be possible to apply techniques from the field of variational analysis to determine the set of optimal solutions, our main objective here is to apply Algorithm 1 without exploiting any particular knowledge about the solution set. For this, we use the Legendre polynomials as basis functions in $L_2[0,T]$,

$$\begin{aligned} \forall i\in \mathbb N \quad \varPhi _i(t) = (-1)^i \sum _{j=0}^i \left( \begin{array}{c}i\\ j\end{array}\right) \left( \begin{array}{c}i+j\\ j\end{array}\right) \left( -\frac{t}{T}\right) ^j, \end{aligned}$$

which are orthogonal by construction.

We start by showing that the functional *F* is strongly Lipschitz-continuous, with the bounded regular subset *G* in condition (7) taken as

$$\begin{aligned} G \,:=\, \left\{ \left. \; f_1^t \; \right| \; t \in [0,T] \; \right\} \cup \left\{ \left. \; f_2^t \; \right| \; t \in [0,T] \; \right\} \; \subseteq \; H \; , \end{aligned}$$

where we use the shorthand notation $f_1^t(\tau ) := f_1(t - \tau )$ and $f_2^t(\tau ) := f_2(t -\tau )$. For all $x\in L_2[0,T]$ and all $e\in H$, we have

$$\begin{aligned} \left| F(x+e) - F(x) \right|= & {} \left| \int _0^T \langle f_1^t , x+e \rangle ^2 - \langle f_1^t , x \rangle ^2 - \langle f_2^t , x+e \rangle ^2 + \langle f_2^t , x \rangle ^2 \, \mathrm {d}t \right| \\= & {} \left| \int _0^{T} \langle f_1^t , 2x+e \rangle \langle f_1^t , e \rangle - \langle f_2^t , 2x+e \rangle \langle f_2^t , e \rangle \, \mathrm {d}t \right| \\\le & {} L \, \max \left\{ \sup _{t \in [0,T]} \left| \langle f_1^t , e \rangle \right| , \sup _{t \in [0,T]} \left| \langle f_2^t , e \rangle \right| \right\} = \sup _{g \in G} \, \left| \langle g, e \rangle \right| \,, \end{aligned}$$

where *L* is any upper bound on the term

22 $$\begin{aligned}&\int _0^{T} \left| \langle f_1^t , 2x+e \rangle \right| +\ \left| \langle f_2^t , 2x+e \rangle \right| \, \mathrm {d}t \nonumber \\&\quad \le 2 \int _0^{T} \max _{\tau \in [0,T]} \left( \left| f_1^t(\tau ) \right| + \left| f_2^t(\tau ) \right| \right) \, \mathrm {d}t + \; 2 T \, \sup _{g \in G} \, \left| \langle g, e \rangle \right| \nonumber \\&\quad \le T \left( 22 + \frac{\pi }{2} + 2 \, \sup _{g \in G} \, \left| \langle g, e \rangle \right| \right) \; . \end{aligned}$$

In order to obtain an explicit bound, we need to further analyze the term $\sup _{g \in G} \, \left| \langle g, e \rangle \right|$. First of all, we have

$$\begin{aligned} \overline{D}_C(M) \;\le \; \gamma \;=\; \sup _{x \in C} \Vert x \Vert _2 \;=\; \sqrt{T} \, . \end{aligned}$$

Next, recalling that the Legendre approximation error for any smooth function $g \in L_2[0,T]$ is bounded as

$$\begin{aligned} D(M,g) := \left\| g - P_M(g) \right\| _2 \;\le \; \frac{\mu _{M+1} \sqrt{T}}{(M+1)!} \left( \frac{T}{M} \right) ^{M} \quad \text {with} \quad \mu _i := \sup _{\xi \in [0,T]} \left| \frac{ \partial ^i g}{ \partial t^i }(\xi ) \right| \end{aligned}$$

for all $M \ge 1$, and working out explicit bounds on the derivatives of the functions $f_1^t$ and $f_2^t$, we obtain

$$\begin{aligned} \forall M\in \mathbb {N}^+, \quad \sup _{g \in G} D(M,g)\le & {} \frac{T^{\frac{3}{2}}}{(M+1)!} \left( \frac{T}{M} \right) ^{M} \left( \frac{1}{2}+\frac{M}{\pi } \right) \left( \frac{\pi }{2T} \right) ^{M} \\\le & {} \frac{3}{4} \frac{T^{\frac{3}{2}}}{(M+1)!} \left( \frac{\pi }{2M} \right) ^{M-1} \, . \end{aligned}$$

It follows by Theorem 1 that

$$\begin{aligned} \sup _{g \in G} \, \left| \langle g, e \rangle \right| \; \le \; \overline{R}_C(M,G) \; = \; \sup _{g \in G} \overline{D}_C(M) D(M,g) \; \le \; \frac{3}{4} \frac{T^2}{(M+1)!} \left( \frac{\pi }{2M} \right) ^{M-1} \, . \end{aligned}$$

Combining all the bounds and substituting $T = 10$ shows that the constant $L = 611$ satisfies the condition (22).

Based on the foregoing developments and the considerations in Sect. 3.2, a simple bound $\varDelta _M(A)$ on the approximation error satisfying (17) can be obtained as

$$\begin{aligned} \forall (M,A) \in \mathbb {N}^+\times \mathbb S^{M+1}, \quad&\varDelta _M(A) = \frac{45825}{(M+1)!} \left( \frac{\pi }{2M} \right) ^{M-1} \, . \end{aligned}$$

Although rather loose for very small *M*, this estimate converges quickly to 0 for larger *M*; for instance, $\varDelta _7(A) \le 2 \cdot 10^{-4}$. Note also that, in a practical implementation, the computation of $\varDelta _M(A)$—and also to validate the generalized Lipschitz constant *L*—could be automated using computer algebra programs, such as Chebfun (http://www.chebfun.org/) [16] or MC++ (https://github.com/omegaicl/mcpp) [35].

With regards to the computation of bounds $L^0_M(A)$ and $U^0_M(A)$ satisfying (16), we note that *F*(*x*) can be interpreted as a quadratic form in *x*,

$$\begin{aligned} F \left( \sum _{i=0}^M a_i \varPhi _i \right) \; = \; a^\mathsf {T}Q a \; , \end{aligned}$$

with the elements of the matrix *Q* given by

$$\begin{aligned} \forall j,k \in \{ 0, \ldots , M \}, \quad Q_{j,k} \; = \; \int _0^T \left( \langle f_1^t , \varPhi _j \rangle \langle f_1^t , \varPhi _k \rangle - \langle f_2^t , \varPhi _j \rangle \langle f_2^t , \varPhi _k \rangle \right) \, \mathrm {d}t \; . \end{aligned}$$

Of the available approaches [18, 39, 41] to compute bounds $L^0_M(A)$ and $U^0_M(A)$ such that

$$\begin{aligned} L^0_M(A) \; \le \; \min _{a \in A} \, a^\mathsf {T}\, Q \, a \; \le \; U^0_M(A) \end{aligned}$$

for interval boxes $A \subseteq \mathbb R^{M+1}$, we use standard LMI relaxation techniques [20] here.

**Fig. 1:**
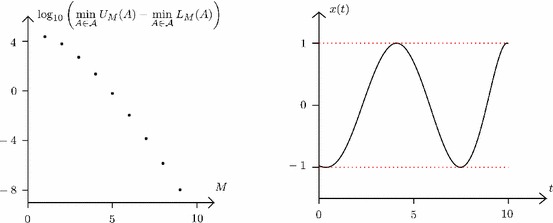
Results of Algorithm 1 applied to Problem (21) for $\varepsilon = 10^{-5}$ and $\rho =1$. Left gap between upper and lower bounds as a function of the lifted subspace dimension *M*. Right a globally $\varepsilon$-suboptimal solution *x*

At this point, we have all the elements needed for implementing Algorithm 1 for Problem (21). On selecting the termination tolerance $\varepsilon = 10^{-5}$ and the lifting parameter $\rho =1$, Algorithm 1 terminates after less than 100 iterations and applies 8 lifting operations (starting with $M=1$). The corresponding decrease in the gap between upper and lower bounds as a function of the lifted subspace dimension *M*—immediately after each lifting operation–is shown on the left plot of Fig. 1. Upon convergence, the infimum of (21) is bracketed as

$$\begin{aligned} -\,\,0.16812 \; \le \; \inf _{x \in C} \; F(x) \; \le \; -0.16811 \; , \end{aligned}$$

and a corresponding $\varepsilon$-global solution *x* is reported on the right plot of Fig. 1; the symmetric function $(-\,\,x)$ provides another $\varepsilon$-global solution for this problem. Overall, this case study demonstrates that the proposed branch-and-lift algorithm is thus capable of solving such non-convex and infinite-dimensional optimization problem to global optimality within reasonable computational effort.

## Conclusions

This paper has presented a complete-search algorithm, called branch-and-lift, for global optimization of problems with a non-convex cost functional and a bounded and convex constraint sets defined on a Hilbert space. A key contribution is the determination of run-time complexity bounds for branch-and-lift that are independent of the number of variables in the optimization problem, provided that the cost functional is strongly Lipschitz-continuous with respect to a regular and bounded subset of that Hilbert space. The corresponding convergence conditions are satisfied for a large class of practically relevant problems in calculus of variations and optimal control. In particular, the complexity analysis in this paper implies that branch-and-lift can be applied to solve potentially non-convex and infinite-dimensional optimization problems without needing a-priori knowledge about the existence or regularity of minimizers, as the run-time bounds solely depend on the structural and regularity properties of the cost functional as well as the underlying Hilbert space and the geometry of the constraint set. This could pave the way for a new complexity analysis of optimization problems, whereby the “complexity” or “hardness” of a problem does not necessarily depend on their number of optimization variables. In order to demonstrate that these algorithmic ideas and complexity analysis are not of pure theoretical interest only, the practical applicability of branch-and-lift has been illustrated with a numerical case study for a problem of calculus of variations. The case study of an optimal control problem in [25] provides another illustration.

## Footnotes

1 We have used the integration formula $\displaystyle \int e^{\sqrt{a x}} \, \mathrm {d}x = \frac{2 e^{\sqrt{ax}}(\sqrt{ax}-1)}{a} + C$ for the integral term in (6).
